# Effects of Neoadjuvant Intraperitoneal/Systemic Chemotherapy (Bidirectional Chemotherapy) for the Treatment of Patients with Peritoneal Metastasis from Gastric Cancer

**DOI:** 10.1155/2012/148420

**Published:** 2012-07-31

**Authors:** Yutaka Yonemura, Ayman Elnemr, Yoshio Endou, Haruaki Ishibashi, Akiyoshi Mizumoto, Masahiro Miura, Yan Li

**Affiliations:** ^1^NPO Organization to Support Peritoneal Surface Malignancy Treatment, Osaka, Kishiwada 596-0032, Japan; ^2^Department of Surgery, Kusatsu General Hospital, Shiga, Kusatsu 525-8585, Japan; ^3^Department of Surgery, Peritoneal Surface Malignancy Center, Kishiwada Tokushukai Hospital, Kishiwada 596-8522, Japan; ^4^Peritoneal Dissemination Program, Kishiwada Tokushukai Hospital and Kusatsu General Hospital, NPO Organization to Support Peritoneal Surface Malignancy Treatment, 1-26, Haruki-Moto-Machi, Osaka, Kishiwada City, 596-0032, Japan; ^5^Department of Surgery, Tanta University Hospital, Tanta, Egypt; ^6^Department of Experimental Therapeutics, Cancer Research Institute, Kanazawa University, Kanazawa 920-1192, Japan; ^7^Department of Anatomy, School of Medicine, Oita University, Oita 870-1192, Japan; ^8^Department of Oncology, Zhongnan Hospital, Cancer Center of Wuhan University, Wuhan 430072, China

## Abstract

Novel multidisciplinary treatment combined with neoadjuvant intraperitoneal-systemic chemotherapy protocol (NIPS) and peritonectomy was developed. Ninety-six patients were enrolled. Peritoneal wash cytology was performed before and after NIPS through a port system. Patients were treated with 60 mg/m^2^ of oral S-1 for 21 days, followed by a 1-week rest. On days 1, 8, and 15, 30 mg/m^2^ of Taxotere and 30 mg/m^2^ of cisplatin with 500 mL of saline were introduced through the port. NIPS is done 2 cycles before surgery. Three weeks after NIPS, 82 patients were eligible to intend cytoreductive surgery (CRS) by gastrectomy + D2 dissection + periotnectomy to achieve complete cytoreduction. Sixty-eight patients showed positice cytology before NIPS, and the positive cytology results became negative in 47 (69%) patients after NIPS. Complete pathologic response on PC after NIPS was experienced in 30 (36.8%) patients. Stage migration was experienced in 12 patients (14.6%). Complete cytoreduction was achieved in 58 patients (70.7%). By the multivariate analysis, complete cytoreduction and pathologic response became a significantly good survival. However the high morbidity and mortality, stringent patient selection is important. The best indications of the therapy are patients with good pathologic response and PCI ≤ 6, which are supposed to be removed completely by peritonectomy.

## 1. Introduction

In the past, peritoneal carcinomatosis (PC) from gastric cancer has been regarded as a terminal stage [1], and the most oncologists regarded as a condition only to be palliated. Preusser et al. published a response rate to chemotherapy of 50% of patients with stage IV gastric cancer, but the response rate was the lowest in patients with PC [2]. Ajani et al. reported that PC was the most common indication of failure of the intensive chemotherapy [3]. Accordingly, surgery alone or chemotherapy alone is not an adequate management for gastric cancer patients with PC.

Over the past two decades, a new multimodal treatment called cytoreductive surgery (CRS) [4] plus perioperative chemotherapy (POC) was proposed. POC includes neoadjuvant chemotherapy (NAC), hyperthermic intraperitoneal chemotherapy (HIPEC), and/or early postoperative intraperitoneal chemotherapy (EPIC), which takes the advantage of surgery to reduce the visible tumor burden and POC to eradicate peritoneal micrometastasis and peritoneal free cancer cells (PFCCs) [5]. Survival analyses after CRS plus HIPEC showed that complete cytoreduction is associated with survival improvement [5, 6]. Neoadjuvant chemotherapy is proposed to reduce tumor burden before operation, resulting in the improvement of the incidence of complete cytoreduction [5]. A new bidirectional chemotherapy (neoadjuvant intraperitoneal-systemic chemotherapy protocol (NIPS)) was developed to induce a reduction of the peritoneal cancer index of PC and to eradicate PFCCs [5]. NIPS can attack PC from both sides of peritoneum, not only from the peritoneal cavity but also from the subperitoneal blood vessels. Accordingly, NIPS is called as bidirectional chemotherapy.

In the present study, the effects of NIPS on the intraperitoneal cytological status, histological response of PC, the incidence of complete cytoreduction, and survivals in the patients with established PC form gastric cancer will be reported.

## 2. Patients and Methods

### 2.1. Patients

Ninety-six patients with primary gastric cancer with PC were enrolled in the study. Enrolled patients in the study were treated between April 2004 and December 2011. PC was diagnosed by biopsy under laparotomy, laparoscopy, or by the cytologic examination of ascites. The eligibility criteria included (1) histologically or cytologically proven PC from gastric adenocarcinoma; (2) absence of hematogenous metastasis and remote lymph node metastasis; (3) age 75 years or younger; (4) Eastern Clinical Oncology Group scale of performance status 2 or less; (5) good bone marrow, liver, cardiac, and renal function; (6) absence of other severe medical conditions or synchronous malignancy.

Informed consent according to the institutional guideline was obtained from all patients.

### 2.2. Methods to Introduce a Peritoneal Port System and Peritoneal Wash Cytology

A peritoneal port system (Hickman subcutaneous port; Bard, Salt Lake City, USA) was introduced into the abdominal cavity under local anesthesia, and the tip of the system was placed on the cul-de-sac of Douglas. Then, a peritoneal wash cytology was performed after 500 mL of physiological saline had been injected into the peritoneal cavity. To improve the accuracy of the cytology, an immunohistochemical examination using monoclonal antibodies for anti-human carcinoembryonic antigen (TAKARA Bio INC., Tokyo, Japan) and anti-human epithelial antigen (DAKO, Copenhagen, Denmark) were performed. A peritoneal wash cytological examination was performed before and after NIPS.

### 2.3. Bidirectional Chemotherapy

Patients were treated with 60 mg/m^2^ of oral S-1 (Taiho Pharmaceutical Co., Ltd., Tokyo, Japan) for 21 days, followed by a 1-week rest. On days 1, 8, and 15 after the start of oral S-1 administration, 30 mg/m^2^ of Taxotere and 30 mg/m^2^ of cisplatinum with 500 mL of saline were introduced through the port. This regimen was repeated after a one-week rest [7]. Bidirectional chemotherapy is done 2 cycles before surgery. The aims of NIPS are to reduce the peritoneal surface involved by PC and to eradicate PFCCs. Toxicities were graded using the CTCAE v 3.0.

### 2.4. Selection Criteria of Patients for Cytoreductive Surgery (CRS) after NIPS

After two cycles of NIPS, patients who had the following criteria are excluded as the candidates for CRS: (1) evidence of para-aortic lymph node involvement and distant hematogenous metastasis confirmed by computed tomography (CT), or magnetic resonance imaging (MRI), (2) patients with progressive disease after NIPS, or (3) patients with severe comorbidities or poor general condition.

### 2.5. Quantitative Evaluation of the Volume of PC and Assessment Completeness of Cytoreduction

**Figure 1:**
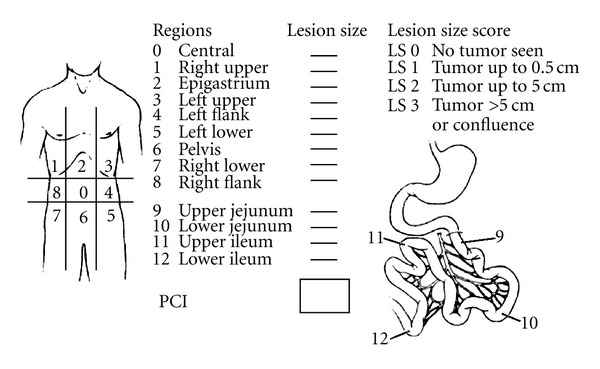
Peritoneal cancer index (PCI). Peritoneal cavity is divided into 13 parts, which ranges from 0 to 12. Accurate measurement of each region is scored as lesion size 0 through 3. LS 0: no implants.

Intraoperatively, the tumor volume was quantified according to the Japanese general rules for gastric cancer study [8] and the Sugarbaker's peritoneal cancer index (PCI) [9]. The abdomen and pelvis were divided into nine regions and the small bowel into four each assigned a lesion size (LS) score of 0–3, representative of the largest implant visualized. LS-0 denotes the absence of implants, LS-1 indicates implants *˂*0.25 cm, LS-2 implants between 0.25 and 5 cm, and LS-3 implants >5 cm or a confluence of disease. These figures amount to a final numerical score of 0–39 (Figure 1).

The aim of CRS was to obtain complete macroscopic cytoreduction as a precondition for the application of HIPEC. The residual disease was classified intraoperatively using the completeness of cytoreduction (CC) score [9]. CC-0 indicates complete cytoreduction with no residual macroscopic nodule; CC-1 indicates no macroscopic tumor but a positive histological margin on the esophageal, duodenal stump, or suspicious residual nodules less than 5 mm in diameter, CC-2 indicates apparent macroscopic residual tumors greater than 5 mm but upto 5 cm in diameter, and CC-3 indicates residual PC greater than 5 cm in diameter.

### 2.6. Methods of CRS Using Peritonectomy Techniques [6]

Laparotomy was done 3 weeks after the last day of NIPS. Under general anesthesia, midline incision was made from the xiphoid to the pubis. Just after laparotomy, peritoneal wash cytology is done, and PCI score was calculated in each case.

For the tissue dissection, electrosurgical techniques are used. In electrosurgery, a generator delivers high-frequency current greater than 200 kHz under high-power electricity (100 Watt), using the electrosurgical generator (Valleylab Inc., Boulder, CO, USA). The mainly used handpiece is the ball-tipped type. The 2 mm ball-tip electrode is used for dissecting on visceral surfaces.

After the left lobe of liver is freed from the left triangular ligament, resection of the lesser omentum along the Arantius duct is started. Gastrectomy combined with D2 dissection [8], greater omentectomy, splenectomy, and the resection of anterior leaf of mesocolon is done. Importantly, the small bowel should be intact for the safe reconstruction either by esophagojejunostomy or gasrojejunostomy. The aim of peritonectomy is to remove all the macroscopic PC nodules with peritoneum. If the parietal peritoneum is involved, both sides of the parietal peritoneum are peeled off from the posterior rectus sheath to the retroperitoneal space. The dissection continues deeply and in a counterclockwise direction, starting in the right flank till reaching the peritoneum covering the left copula of the diaphragm. Then, the dissection is completed in the upper right side till reaching the anterior renal fascia, inferior vena cava, and posterior wall of the duodenum.

The peritoneum of the Morrison's pouch and paracolic gutters on both sides are completely freed from retroperitoneum and is removed with the anterior parietal peritoneum.

If the undersurface of the diaphragm is involved, stripping of peritoneum from the right and left hemidiaphragm is done. The falciform and round ligament are taken down and resected completely.

Large PC nodules attach on the transverse colon are removed in combination with extended right hemicolectomy.

The entire pelvic peritoneum is dissected from the anterior inferior abdominal wall, urinary bladder, and retroperitoneum. The peritoneum covering the urinary bladder is dissected and the rectovesical pouch is completely freed from the urinary bladder and rectum. In male, the space between seminal vesicle and peritoneum of rectovesical pouch is dissected, lifting the vas deferens off. In female, blood vessels around the uterus are dissected and cut with LigaSure (Valleylab Inc., Boulder, CO, USA). Amputation of vagina is done at a plane 1 cm below the peritoneal reflection of Douglas pouch to ensure removal of all tumor occupy the cul-de-sac.

If the tumor invades into the anterior rectal wall, rectum is cut at 1 cm below the peritoneal reflection. Reasonable length of the rectum should be preserved for the anastomosis with the colon.

The entire small bowel and its mesentery are traced from the duodenojejunal flexure to the ileocecal junction. Then, both sides of the mesentery are inspected and palpated, and the tumor nodules excised with electrosurgery. Complete cytoreduction is aimed by removing all macroscopic tumors by peritonectomy combined with electric fulguration.

### 2.7. Histologic Evaluation of NIPS

Histologic effects on primary tumors and PC were evaluated according to the general rules for gastric cancer treatment [8]. Histological response after chemotherapy is classified into 4 categories. Ef-0 shows no histologic response or response less than one-third of the tumor tissue. A histologic Ef-1 means that the degeneration of cancer is detected in the tumor tissue raging from one-third to less than two-thirds of the tumor tissue. EF-2 shows the degeneration of cancer tissue in wider than two-thirds of the tumor tissue, while an Ef-3 means the complete disappearance of the cancer cells.

### 2.8. Statistical Analyses

All patients were followed and no patients were lost to follow up. Outcome data were obtained from medical records and patients' interview. All statistical analyses had performed using SPSS software statistical computer package version 17 (SPSS Inc., Chicago, USA).

## 3. Results

Clinical characteristics of the 96 patients are listed in Table 1.

The average age was 51.3 years, including 42 men and 54 women. All 96 patients had primary gastric cancer and had P2 or P3 dissemination. Ascites was found in 55 (57%) patients.

Before NIPS, cytology had been positive in 68 (70.8%) of 96 patients and was positive in 21 (22.9%) after NIPS. These 68 positive cytology results before NIPS became negative in 47 (69%) patients after NIPS (Table 2).

After NIPS, 82 patients received operation, and the other 14 patients did not undergo operation due to the progression of disease or refusal of operation. At laparotomy, P status in Japanese rules became to be P0 in 7, P1 in 11, P2 in 8, and P3 in 56 patients. Mean PCI was 6.3, ranging from 0 to 33, and PCI ≤ 6 and PCI ≥ 7 were 56 and 26 patients, respectively.

Table 3 indicates the operation methods. Total gastrectomy was performed in 67 patients. A variety of supplemental procedures were performed to achieve tumor cytoreduction. The common procedures for visceral peritonectomy were transverse colectomy combined with right hemicolectomy and omentectomy (*N* = 33), pelvic peritonectomy in 38 including low anterior resection in 17, bilateral salpingo-oophorectomy in 36 of 48 female patients, segmental resection of small bowel, and small-bowel mesentery in 18 and 16 patients. Left and right subdiaphragmatic peritonectomy was performed in 25 and 22 patients, respectively. Mean operation time was 230 min (120~690 min), and mean blood loss was 1571 mL (850~4540 mL).

Complete cytoreduction (CC-0) was achieved in 58 of 82 patients (70.7%). CC-0 was achieved in 48 (78.7%) of 61 patients with negative cytological status after NIPS, but was done in 10 (47.6%) of 21 patients with positive cytology after NIPS. Regarding the PCI, CC-0 was done in 54 (96.4%) of 56 patients with PCI score ≤ 6, but was performed in10 (38.4%) of 26 patients with PCI ≥ 7. Causes of incomplete cytoreduction were diffuse involvement of small bowel in 7 patients, PCI score higher than 20 in 6 patients, positive margin on esophageal stump in 3 patients, and local invasion into the retroperitoneal tissue in one patient.

During NIPS, side effects of level 3, 4, and 5 were found in 7 (7.3%), 2 (2.1%), and 1 (1.0%) patients (Table 4). One patients died of aspiration pneumonia due to ileus (grade 5).

After NIPS and CRS, 8, 9, and 3 patients developed grade 3, 4, and 5 complications (Table 5). The most frequent complications are anastomosis dehiscence. The overall operative mortality rate was 3.7% (3/82), and the cause of death was multiple organ failure with renal failure, hepatic coma, and sepsis due to anastomosis leakage. Grade 4 complications were found in 9 patients, and three patients developed renal failure were treated by hemodialisis. Six patients underwent operation for the postoperative bleeding in one patient, drainage of the abscess from anastomotic leakage in four patients, and port infection in one patient. All 8 patients developed grade 3 complications recovered well after appropriate treatments.

**Figure 2:**
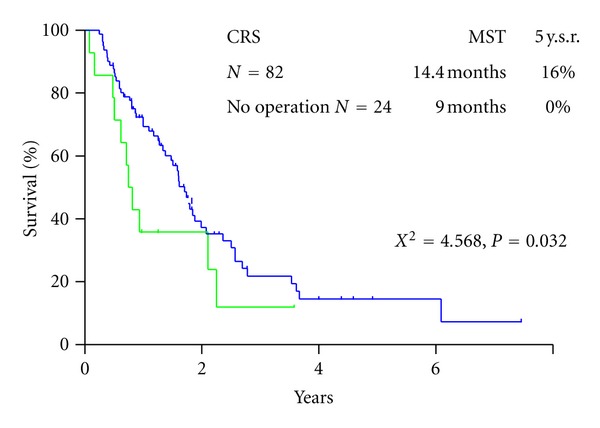
Survivals of 82 patients who underwent CRS, and 14 patients who did not underwent CRS.

**Figure 3:**
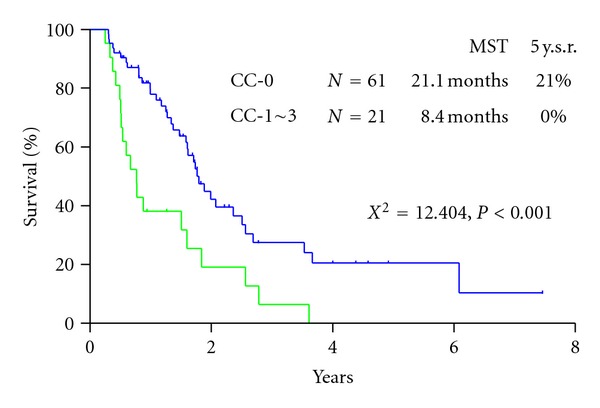
Survival difference after CC-0 and CC-1 resection.

**Figure 4:**
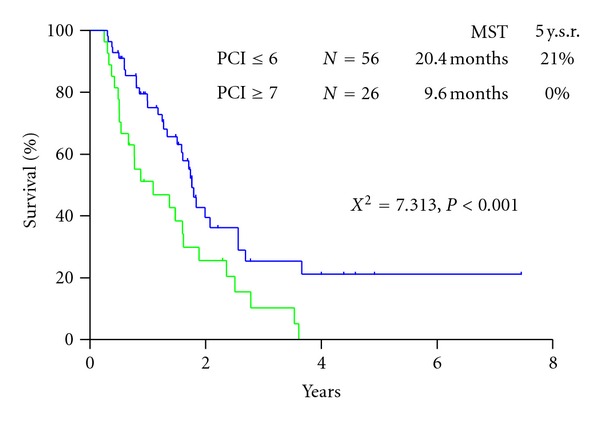
Survival difference of patients with PCI ≤ 6 and those with PCI ≥ 7.

Twenty-six (31.7%) were alive at the time of analysis. The survival curve for all patients is shown in Figure 2. Median survival time (MST) of patients who underwent CRS was 14.4 months, with a one-, three-, and five-year survival of 61%, 16%, and 16%, respectively. MST of patients who did not receive operation was 9.0 months (Figure 2). There was a significant survival difference between the two groups (*P* < 0.05). Patients who received a complete resection (CC-0) had an MST of 21.1 months, and MST of patients who received incomplete cytoreduction (CC-1~3) was 8.4 months (*P* < 0.001) (Figure 3). Significant survival difference was found between CC-0 and CC-1~3 group. MST of patients with PCI ≤ 6 was 20.4 months with a 5-year survival of 21.0% and that of patients with PCI ≥ 7 was 9.6 months with no 5-year survival. There was a significant survival difference between the two groups (*P* < 0.001) (Figure 4).

**Figure 5:**
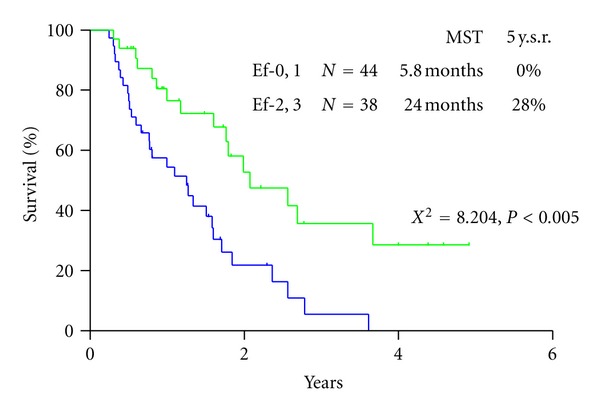
Survival difference of patients, according to the histological response on PC after NIPS.

Histologic effects on primary tumors were found in 71 of the 82 tumors, and Ef-1, -2, -3 response in the primary tumors were detected in 43 (52.5%), 28 (34.1%), and 0 tumor (0%), respectively (Table 6). In contrast, the complete histologic disappearance of PC was observed in 30 (36.8%) of 82 patients (Table 6). Stage migration from stage 4 to stage 1, 2, or 3 was experienced in 2, 2, and 8 patients. MST of patients with Ef-0/Ef-1 effects in PC tissue was 5.8 months with a 5-year survival of 0% and that of patients with Ef-2/Ef-3 effects was 24.0months with a 5-year survival of 28.0% (Figure 5). There was a significant survival difference between the two groups (*P* < 0.001).

As shown in Table 7, among various prognostic factors, CC score and pathologic effects were independent prognostic factors.

Recurrence was found in peritoneum, lung, liver hilum, liver, and bone in 27, 5, 3, 2, and 2 patients, respectively.

## 4. Discussion

 The current state-of-the-art treatment for colorectal peritoneal dissemination consists of a comprehensive management strategy using CRS and POC [10]. Patients with a low tumor volume, well/moderately differentiated tumors, and complete cytoreduction may potentially benefit from combined treatment. In gastric cancer patients with PC, no survival benefit has been reported by cytoreduction alone [1]. In contrast, CRS with peritonectomy plus HIPEC confers a prolonged survival period [5]. Furthermore, complete cytoreduction is an essential factor for a good outcome, and NIPS plus peritonectomy may improve the incidence of complete cytoreduction [7]. The aims of neoadjuvant chemotherapy (NAC) are stage reduction, eradication of micrometastasis outside the surgical field, and the improvement of resectability. Systemic chemotherapy usually is used for NAC. In the late 1990s, TS-1, irinotecan, taxanes, and docetaxel were introduced for gastric cancer treatment, and the response rates after monotherapy with these drugs were around 20%. Combination chemotherapy with S-1 and CDDP produced outstanding results, with a response rate of 74% [11]. Yabusaki et al. reported the results of NAC with S-1 and CDDP in 37 advanced gastric cancer patients. After 2 courses of treatment, the overall response rate was 68%, but the response rate for patients with peritoneal dissemination was only 14% (2/14) [12]. S-1 plus CPT-11 and CPT-11 plus CDDP produced a high-response rate of 42% and a long period of progression-free survival, but treatment failure as a result of toxicity was also observed [13].

These results indicated that systemic chemotherapies have minimal effects on PC. In other words, the peritoneal cavity acts as a sanctuary against systemic chemotherapy, probably because of the existence of a blood-peritoneal barrier consisting of stromal tissue between mesothelial cells and submesothelial blood capillaries [9, 14]. This barrier accounts for a total thickness of 90 *μ*m [14]. Accordingly, only a small amount of systemic drugs are capable of penetrating this barrier and passing into the peritoneal cavity, and a higher percentage of the administered drugs instead moves to the bone marrow and vital organs other than the peritoneum, resulting in the development of adverse effects.

In contrast, IP chemotherapy offers potential therapeutic advantages over systemic chemotherapy by generating high local concentrations of chemotherapeutic drugs in the peritoneal cavity [15, 16]. This advantage of IP chemotherapy can be expressed by the area under the curve (AUC) ratios of intraperitoneal versus plasma exposure.

Relatively high AUC IP/IV ratios were obtained after the IP administration of paclitaxel, docetaxel, gemcitabine, 5-fluorouracil, and doxorubicin [16]. These drugs may be good candidates for IP chemotherapy.

Other important factors in the selection of drugs for IP chemotherapy are a high-penetration activity into the PC nodules and chemosensitivity. Cisplatin and carboplatin have the highest penetration activity and were confirmed to penetrate 1 to 2 mm from the surface of PC nodules [15]. In an experimental PC model using a highly metastatic cell line derived from human gastric cancer, docetaxel, 5-FU, carboplatin, and TS-1 plus cisplatin was highly effective for improving the survival of nude mice bearing PC, and the IP administration of these drugs is expected to become a standard therapy for gastric cancer patients [17, 18].

From these clinical and experimental results, a new bidirectional chemotherapy combined with the oral administration of S-1 and IP CDDP and docetaxel has been developed. By simultaneously administering intravenous and intraperitoneal chemotherapy, a bidirectional diffusion gradient can create a wider treatment area than single treatment.

The Cox multivariate analysis clearly demonstrated the complete cytoreduction and pathological response after NIPS were the independent prognostic factors. The factors to achieve CC-0 resection correlated with the negative cytology after NIPS and PCI score ≤ 6. Furthermore, peritonectomy techniques enable to achieve CC-0 resection even in the patients with higher PCI score [6, 7].

Preoperative evaluation of PC from gastric cancer is limited, and the sensitivity of CT for detecting PC was influenced by the lesion size. Koh et al. reported the value of preoperative CT in estimating PC in patients with colorectal carcinomatosis [19]. The depiction rate of small-bowel involvement had the lowest sensitivity, with a rate of 8–17%., and the false-negative rate significantly decreased with the lesion size. Small nodules (<0.5 cm) were visualized using CT with a sensitivity of 11%, in contrast to a sensitivity of 94% for PC with a diameter greater than 5 cm. Accordingly, preoperative assessment of PCI by CT is not recommended in gastric cancer. In contrast, cytological examination from a port system is very convenient and is an objective evaluation of the intraperitoneal cytologic status. In the present study, CC-0 was achieved in 48 (78.7%) of 61 patients with negative cytological status after NIPS, but was done in 10 (47.6%) of 21 patients with positive cytology after NIPS. There was a significant difference in the incidence of CC-0 resections between the two groups. Accordingly, cytologic examination through a port system may be one of the indicators for CC-0 resection. Furthermore, before NIPS, cytology had been positive in 68 (70.8%) of 96 patients and was positive in 21 (22.9%) after NIPS. These 68 positive cytology results before NIPS became negative in 47 (69%) patients after NIPS. NIPS can eradicate PFCCs before CRS and may prevent the attachment of PFCCS on the surgical wound at CRS.

After systemic neoadjuvant chemotherapy, a complete PC response is very rare. Inokuchi et al. reported that the response rate of PC after S-1 plus irinotecan was 69% 9/13), but no CR was experienced for PC [20]. Baba et al. also reported the limited effects of systemic S-1+CDDP on PC from gastric cancer [21].

After histological examination of the primary tumors and the resected peritoneum, histologic effects on primary tumors were found in 71 of the 82 tumors. However, the Ef-2 response and the complete pathologic response of Ef-3 in primary tumors were found in 28 tumors (34.1%) and 0 tumor (0%). In contrast, those in the peritoneal dissemination were observed in 30 (36.8%) of 82 patients. In addition, stage migration from stage 4 to stage 1, 2, or 3 was experienced in 12 patients (14.6%). These results indicate that NIPS can be a powerful strategy for eradicating PFCCs and for the reduction of the PCI score.

The present study demonstrated that the survival results were significantly better when the PCI was lower than 6. CC-0 resection was done significantly higher in patients with PCI ≤ 6 than PCI ≥ 7. The frequent cause of incomplete cytoreduction was the diffuse involvement of the small bowel. NIPS can reduce PCI score, and the timing of peritonectomy can be determined by the laparoscopic examination. Garofalo reported an excellent experience of laparoscopic diagnosis for PC [22]. There was a good correlation between the open-surgery data and the laparoscopic PCI scores. If the PCI score determined by the laparoscopy is larger than 7 accompanying with small-bowel involvement, NIPS is recommended to reduce PCI score on the small bowel. In contrast, patients with a PCI score >7 even after NIPS should be treated with palliative intent without peritonectomy. The PCI score is believed to be an independent prognostic factor, and a PCI score is capable of serving as a threshold for favorable versus poor prognosis.

NIPS may add to the morbidity and mortality of further surgical treatment [7]. The incidence of major side effects (grade 3, 4, and 5) after NIPS was 10.4% (10/96). Chemotherapy-related death was found in one patient, and she died of aspiration pneumonia due to bowel obstruction without relation with chemotherapy. Renal dysfunction occurred in one patients (1.0%), but the patients recovered fully by hemodialysis. Accordingly, the new bidirectional chemotherapy regimen is considered to be a safe method as compared with the results of previous reports of systemic chemotherapy [2, 3, 23, 24].

The present study demonstrated that NIPS plus peritonectomy may improve the incidence of complete cytoreduction. However, NIPS might increase the risk of a peritonectomy procedure plus a gastrectomy combined with a lymphadenectomy. Glehen reported a mean operation time of 5.2 hours (range, 1.5–9.5 hours), a 30-day mortality rate of 4% (2/49), and a major complication rate of 27% (13/49) [22]. In the present study, three hospital deaths (3.7%) occurred in patients who died of multiple organ failure (MOF) from a pancreatic fistula, anastomotic leakage, and sepsis. Postoperative major complications occurred in 20 (24.4%) patients. A second operation was necessary in 6 patients, who had complications from leakage of esophagojejunal anastomosis, bleeding, ileal and colonic fistulae, and port-site infection. Glehen reported a higher complication rate of 47% in patients who underwent extensive cytoreductive surgery (gastrectomy combined with the removal of more than 2 peritoneal zones) [22]. The magnitude of surgery, the number of resected organs, the number of anastomoses, and the operation time are considered to have contributed to the significantly higher complication rate. To avoid futile aggressive treatments, the stringent selection of patients must be emphasized preoperatively. Surgeons should have not only a large amount of surgical experiences with gastrointestinal and genitourinary diseases, but also an extensive knowledge of organ anatomy and physiology. Also, surgeons must be able to judge the balance between the postoperative risk associated with the magnitude of the peritonectomy and the survival benefit and quality of life after the aggressive treatment.

In conclusion, NIPS and complete cytoreduction are the essential treatment modalities for the improvement of survival of patients with PC from gastric cancer. Surgeons should experience a learning curve with this procedure at the specialized center and should recommend the accumulation of experience to achieve an acceptable morbidity rate.

## Figures and Tables

**Table 1:** Clinicopathologic characteristics of 96 primary gastric cancer patients with PC.

	Results	CRS (*N* = 82)	No operation (*N* = 14)
Age, years (median)	25–76 (51.3)	25–74 (52.2)	28–75 (46.9)
Gender (male/female)	42/54	34/48	8/6
Histologic type			
Differentiated		3	1
Poorly differentiated		79	13
Lymph node metastasis			
pN0		16	
pN1		40	
pN2		17	
pN3		9	
Macroscopic type			
Type 3		16	3
Type 4		66	11
Liver metastasis	0	0	0
Completeness of cytoreduction			
Complete cytoreduction (CC-0)		61	
Incomplete cytoreduction (CC-1~3)		21	
Hyperthermic intraoperative chemotherapy (HIPEC)			
Done		53	
Not done	29

**Table 2:** Changes of peritoneal lavage cytology before and after NIPS.

Wash cytology after NIPS			
Wash cytology before NIPS	Negative	Positive	Total
Negative	27	1	28
Positive	47	21	68 (70.8%)

74	22 (22.9%)	96

**Table 3:** Surgical procedures for CRS.

Surgical procedures	Patients number
Gastrectomy	
Total gastrectomy	67
Subtotal distal gastrectomy	15
Resection of right diaphragmatic copula	22
Resection of left diaphragmatic copula	25
Greater omentectomy	82
Pelvic peritonectomy	38
Hysterectomy	34/48
Salpingo-oophorectomy	36/48
Right hemicolectomy	33
Low anterior resection	17
Small-bowel resection	18
Resection of small-bowel mesentery	16

**Table 4:** Adverse effects after NIPS.

Side effects	Grade 3	Grade 4	Grade 5
Hematological			
Leukopenia	1 (1.0%)	0	0
Thrombocytopenia	1 (1.0%)	1 (1.0%)	0
Nonhematological			
Stomatitis	0	0	0
Diarrhea	1 (1.0%)	0	0
Nausea, vomiting	1 (1.0%)	0	1 (1.0%)
Fatigue	2 (2.1%)	1 (1.0%)	0
Renal function (need hemodialysis)	1 (1.0%)	0	0

7 (7.3%)	2 (2.1%)	1 (1.0%)

**Table 5:** Complications after NIPS and CRS.

Complications			
Grade 1-2	Grade 3	Grade 4	Grade 5
*N* = 4 (4.9%)	*N* = 8 (9.8%)	*N* = 9 (11.0%)	*N* = 3 (3.7%)
Minor leakage: 2	Pancreas fistula: 2	Renal failure: 3	MOF from leakage: 3
	Major leakage: 4	Major leakage: 4	
Abd. wall dehiscence: 1	Abdominal bleeding: 1	Bleeding: 1	
Abdominal abscess: 1	Port infection: 1

**Table 6:** Pathological response after NIPS.

	Responder			
Ef-0	Ef-1	Ef-2	Ef-3
Primary tumor	11 (13.4%)	43 (52.5%)	28 (34.1%)	0 (0%)
PC	25 (30.4%)	20 (24.3%)	7 (8.5%)	30 (36.8%)

**Table 7:** Prognostic parameters (results of Cox proportional hazard model and logrank test).

	Cox hazard model		Logrank test			
*X* ^ 2^	*P*	Realtive sisk	95% CI	*X* ^ 2^	*P*
Sex (male versus female)	2.158	0.141	0.652	0.3697–1.1531	4.298	0.038
Age (≤65 versus >65)	0.603	0.437	1.399	0.5991–3.2692	0.289	0.59
Histologic type (diff. versus poorly)	0.024	0.876	1.171	0.1584–8.6651	0.631	0.427
CC (CC-0 versus CC-1)	4.197	0.04	2.005	1.0306–3.9041	8.537	0.003
PCI (≥6 versus ≤7)	1.592	0.206	1.528	0.7908–2.9536	5.737	0.017
Pathologic response on PC (Ef-1, -2 versus Ef-2, -3)	4.269	0.038	0.429	0.1927–0.9575	10.303	0.001
LN status (pN0 versus pN1, 2, and 3)	2.478	0.115	2.121	0.8317–5.4108	3.739	0.053
Cytology after NIPS (class I versus class V)	0.047	0.828	1.079	0.5415–2.1513	0.365	0.546
